# Effective Apixaban removal using hemoadsorption during emergent open-heart surgery: a case report and narrative literature review

**DOI:** 10.1186/s13019-024-02748-1

**Published:** 2024-04-06

**Authors:** Vitor Mendes, Jaid Mamode, Jalal Jolou, Mourad Malki, Christoph Ellenberger, Mustafa Cikirikcioglu, Christoph Huber

**Affiliations:** 1 Division of Cardiovascular Surgery, Department of Surgery, Geneva University Hospitals, 1205 Geneva, Switzerland; 2 Department of Anesthesiology, Pharmacology Division of Anesthesiology, Intensive Care and Emergency Medicine, Geneva University Hospitals, Rue Gabrielle-Perret- Gentil 4, 1205 Geneva, Switzerland; 3 Faculty of Medicine, University of Geneva, Geneva, Switzerland

**Keywords:** Cytosorb^®^, Hemoadsorption, Cardiopulmonary bypass, Apixaban, Case report

## Abstract

**Background:**

The management of hemostasis in patients medicated with apixaban (Eliquis) undergoing emergency cardiac surgery is exceedingly difficult. The body’s natural elimination pathways for apixaban prove ineffective in emergency situations, and the impact of hemodialysis is limited. The application of Cytosorb^®^ may attenuate the concentration of apixaban, thereby facilitating the stabilization of these patients.

**Case presentation:**

An 84-year-old man treated with apixaban, underwent emergency ascending aorta replacement surgery due to an acute type A aortic dissection. To address the challenges induced by apixaban, we integrated Cytosorb^®^ cartridge into the Cardiopulmonary bypass circuit. There was a 63.7% decrease in perioperative apixaban-specific anti-factor Xa activity. The patient’s postoperative course was favourable.

**Conclusion:**

Hemoadsorption with Cytosorb^®^ may offers a safe and feasible approach for reducing apixaban concentration in emergency cardiac surgery, thereby mitigating the risk of hemorrhagic complications.

## Background

Cardiopulmonary bypass (CPB) is a technique employed in most cardiac surgery cases. In addition to the underlying disease and the procedure performed, CPB can lead to the development of Systemic Inflammatory Response Syndrome (SIRS), largely mediated by cytokines. Severe cases of SIRS are associated with a prolonged need for vasopressors and mechanical ventilation, and thus, an extended stay in the Intensive Care Unit (ICU) [1].

Hemoadsorption with Cytosorb^®^ (CytoSorbents Corporation, Monmouth Junction, NJ, USA) is a novel therapy that has garnered recent attention due to its association with relief from hyper-inflammatory responses in critically ill patients [2]. Cytosorb^®^ is currently approved by the Swiss Federal Statistical Office, as well as by the European Union and Food and Drug Administration, as an extracorporeal cytokine adsorber designed to mitigate the effects of cytokine storm that can result in inflammation and multiorgan dysfunction [3, 4].

Hemoadsorption with Cytosorb^®^ can be easily administered by a trained perfusionist using an extracorporeal circuit. Its use is indicated in cases of complex cardiac surgery, infective endocarditis, and in patients with multiple comorbidities. Although initially designed to modulate the inflammatory response and eliminate cytokines and other inflammatory mediators from the blood, it has also demonstrated efficacy in removing drugs such as antiplatelet agents (e.g.,ticagrelor) and novels oral anticoagulants (NOACs) (e.g., apixaban) in emergent surgery cases [5, 6].

Apixaban is a factor Xa (FXa) inhibitor used in various clinical conditions, including deep venous embolism and atrial fibrillation [7].

After administration, Apixaban is rapidly absorbed, reaching peak concentrations 3-4 hours following oral administration. Apixaban undergoes various elimination pathways, including metabolism, biliary excretion, and direct intestinal excretion. However, the primary route of elimination is through renal excretion, constituting approximately 27%. The half-life of Apixaban is 12 hours, but it is only achieved when the glomerular filtration rate is within normal range. This duration may be significantly extended in the presence of renal insufficiency [8].

Due to its high affinity for plasma proteins, hemodialysis exerts only a limited impact on apixaban elimination, approximately 20%. Consequently, conventional dialysis-based approaches are not recommended as an effective method for managing apixaban overdose [9]. In the context of cardiac surgery, bleeding is a common complication, and the use of Apixaban is linked to increased surgical morbidity and mortality.

In this paper, we present a case report of the adjuvant use of hemoadsorption with Cytosorb^®^ in a patient with multiple comorbidities and treated with apixaban, who underwent ascending aorta replacement surgery due to a dissecting pseudoaneurysm of the ascending aorta.

## Case presentation

A 84-year-old man (76.5Kg/174cm), treated with apixaban (5mg twice a day) for new-onset atrial fibrillation and known to have heart failure with reduced ejection fraction (HFrEF) of 35% due to amyloidosis and metastatic prostate adenocarcinoma at bone level diagnosed 2 years previously, was admitted to our hospital with NYHA stage III dyspnea and decompensated heart failure.

Blood tests showed high D-dimer levels (4515 ng/ml) and thoracic computed tomography (CT) scan revealed an acute type A aortic dissection.

He was admitted for emergency ascending aorta replacement surgery.

Preoperative transthoracic echocardiography revealed a moderate aortic regurgitation (regurgitant volume- 22.4 ml), with an ascending aorta diameter of 7 cm and a reduced ventricular ejection fraction of 35-40%.

The EuroScore-II pre-operative mortality risk estimate was 24.2 %.

**Fig. 1:**
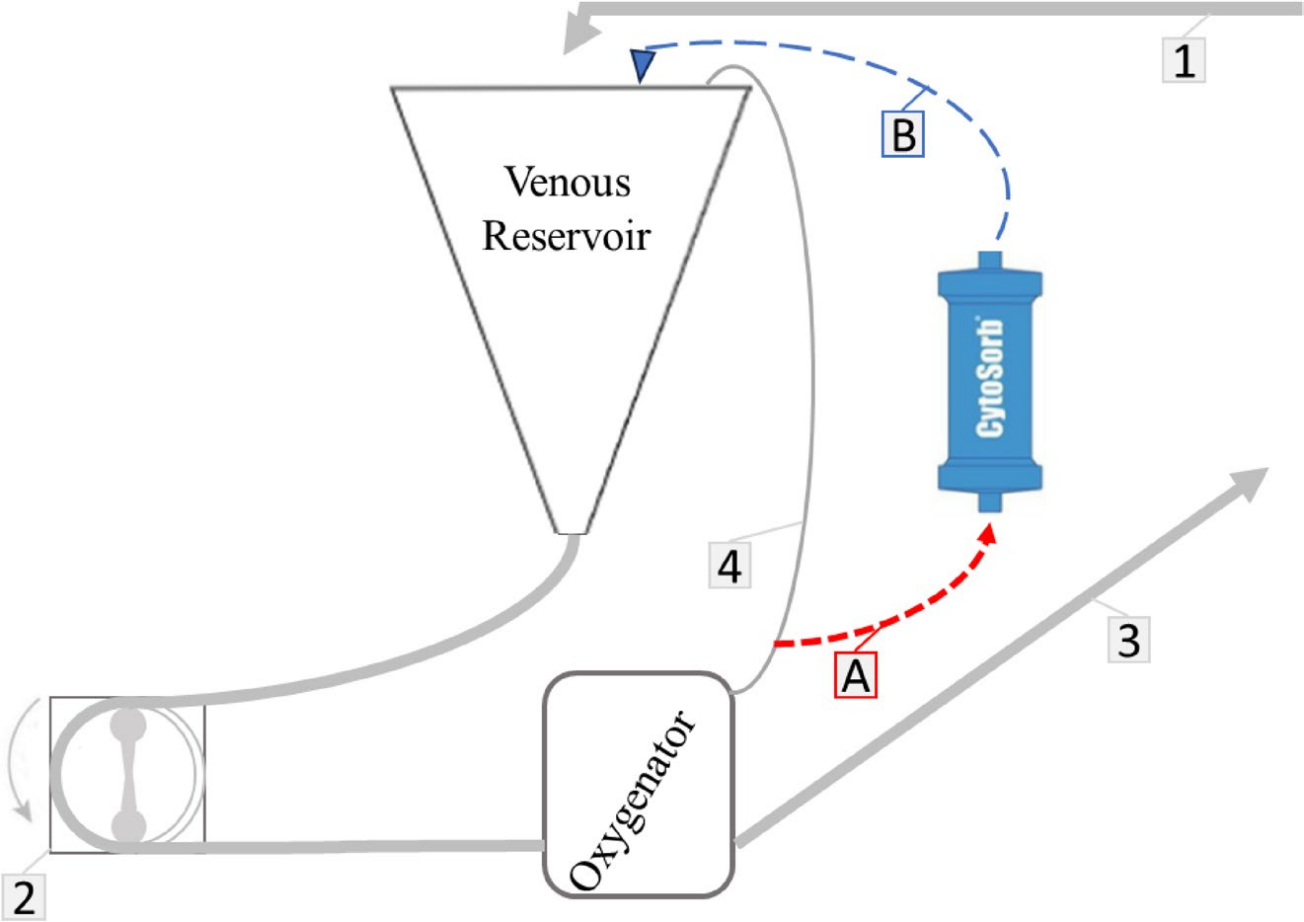
Diagram of Cytosorb^®^ on CPB circuit. 1-Venous line; 2-Roller pump; 3- Arterial line; 4- Recirculation line, A- Aspiration line, B- Infusion line

The CPB circuit included a roller pump, a membrane oxygenator (Affinity Fusion^TM^ , Medtronic, Tolochenaz, Switzerland), a hard-shell reservoir (Affinity Fusion Cardiotomy/ venous Reservoir, Medtronic, Tolochenaz, Switzerland) with a 200 ml minimum operating level, a hemoconcentrator (Dideco DHF02, LivaNova, Sorin Group, Mirandola, Italia) and a cardioplegia device (Myotherm XP^®^, Medtronic, Tolochenaz, Switzerland). A NaCl 0.9% purged CytoSorb^®^ cartridge was inserted between the recirculation line and the venous reservoir (Fig. 1). Hemoadsorption was performed throughout the entire CPB duration.

Preoperatively, the patient presented thrombocytopenia (platelets, 144 g/l), stage II acute kidney failure (serum creatinine, 107 µmol/l). Activated clotting time (ACT) was 195 seconds, Partial thromboplastin time was 33 seconds and apixaban-specific anti-factor Xa activity (AFXaA) was 121 ng/ml (Table 1).

**Table 1:** Laboratory values at Admission in operation room and Intensive Care Unit

	**Admission in OR**	**Admission on ICU**
Hemoglobin (g/l)	139	92
Hematocrit (%)	40.5	27.5
Leukocytes (G/L)	5.1	6.8
Platelets (G/L)	144	78
PTT (s)	40.5	41.5
Fibrinogen (g/l)	2.3	2.7
D-Dimer (ng/ml)	4515	-
Xa-Apixaban activity anti-Xa (apixaban) (ng/ml)	121	44
INR	1.35	1.00
Urea (mmol/l)	8.7	8.1
Creatinine (umol/l)	107	96
eGFR (ml/min/1,73m^2^)	59	68
NT-proBNP (ng/l)	7002	-
Ultrasensitive Troponi-T (ng/l)	69	-
ASAT (U/L)	25	27
ALAT (U/L)	11	17
Alkaline Phosphatase (U/L)	104	56
GGT (U/l)	38	24
Total Bilirubin (umol/l)	40	41

*OR* Operating room, *ICU* Intensive Care Unit, *PTT* Partial Tromboplastin Time, *eGFR* Estimated Glomerular Filtration Rate, *NT-proBNP* N-Terminal pro hormone B-type natriuretic peptide, *ASAT* Aspartate aminotransferase, *ALAT* Alanine Transaminase, *GGT* Gama-Glutamyl Transpeptidase

After administration of 20,000 IU of non-fractioned heparin, the ACT reached 714 seconds. CPB was conducted in standard fashion (ph-stat) at normothermia. The ascending aorta was replaced with a 30 mm diameter Dacron tube. The duration of the surgical intervention was 5h53 with 109 min of CPB duration and 52 minutes of aortic cross-clamping.

After CPB weaning, heparin was reversed with 30,000 IU of protamine and final ACT was 161 seconds. Transoesophageal echocardiography showed a left ventricular ejection fraction of 40% and a discrete aortic regurgitation.

A total of 7 units of packed red blood cells, 2 unit of platelets, 8 units of fresh frozen plasma and 6 gramme of fibrinogen were administered to treat perioperative coagulopathy, as well as 3000 IU of prothrombin complex concentrate (Octaplex^®^) and 5 mg of recombinant factor VIIa (NovoSeven^®^).

The patient was transferred to ICU on 5 ug/kg/min of dobutamine and 0.35 ug/kg/min of norepinephrine. No adverse or any device-related side effects were documented during or after CytoSorb^®^ treatment.

Postoperative chest tube drainage volume at 24h was 280 ml. Dobutamine was discontinued on the end of the first postoperative day, and norepinephrine at the 5^th^ postoperative hour.

The patient was extubated 48h post-surgery, transferred to the ward on the 4^th^ postoperative day and discharged on the 21^st^ postoperative day.

## Discussion

Apixaban is commonly prescribed for the prevention of stroke in patients with non-valvular atrial fibrillation [7]. Patients medicated with apixaban who require emergency surgery face an increased risk of hemorrhagic complications. Andexanet-alfa serves as the antidote to apixaban [10]. In an emergency scenario, this drug can rapidly and almost completely reverse the anticoagulant effects of FXa inhibitors.

However, the effects of andexanet alfa in cardiac surgery and potential interactions with unfractionated heparin are not well-established. Additionally, andexanet-alfa comes at a high cost (US$24,750) [11].

In a cost-utility study aimed at assessing the inherent cost of hemoadsorption in patients treated with ticagrelor, it was found that the use of Cytosorb^®^ could save approximately GBP 4,000 within 30 days. This analysis encompassed the cartridge price, reduced consumption of blood products, and shorter time spent in an emergency surgical environment [5]. Economic factors and ease of use have favoured intraoperative hemoadsorption with Cytosorb^®^ for apixaban elimination in an emergency surgery context [5].

Hemoadsorption with Cytosorb^®^ can be easily integrated into a CPB circuit. In our case, the cartridge was inserted between the recirculation line and the venous reservoir (Fig. 1).

The fundamental principle is that the aspiration line should be connected to a high-pressure area of the circuit (after the arterial pump), and the ejection line should be connected to a low-pressure point in the circuit. This type of connection prevents veno-arterial shunting and gas embolism, allowing for a constant flow in the arterial part of the circuit.

The ideal flow rate through the Cytosorb^®^ cartridge is unknown; however, manufacturers recommend a flow rate between 100-700 ml/min [12]. Recirculation generated through the cartridge should be considered in the blood flow delivered to the patient. As for anticoagulation, it should be mandatory and comprehensive during therapy due to the extensive blood-cartridge contact surface (>40,000m^2^) [13]. Since this therapy involves an extracorporeal circuit, this point may not be an issue. Nevertheless, it is important to note that partial anticoagulation is likely to result in rapid device thrombosis, greatly limiting its effectiveness [13].

To support our findings, we conducted a brief literature review (up until October 1, 2023) on the PubMed database. Only five articles describing interactions between Cytosorb^®^ and apixaban were identified. The key findings are summarized in Table 2.

**Table 2:** Findings of the literature review

**Author’s year**	**Study design**	**Measuring Method**	**Aim Characteristics**	**Results**	**Conclusions**
Røed-Undlien, et al. (2022) [14]	Experimental Study: Laboratory Case-study	In-Vitro	“*Examine if the apixaban concentration in blood could be reduced … by the use of Cytosorb*” -Apixaban concentration was measured at 0,5,15,30,60 and 120 minutes of absorption.	- After 30 minutes, the mean apixaban concentration was reduced from 414.3 (±69.1) ng/mL to 33 (±11.4) ng/mL.	Apixaban concentrations were effectively reduced, and the clotting time and thrombin generation assays were normalized by the use of Cytosorb^®^.
Mendes, et al. (2021) [6]	Observational Study: Case report	In-Vivo	83-Year-old women treated with apixaban underwent emergent redo mitral valve replacement for prothestic valve endocarditis.	- After 100 minutes of CPB, 50% AFXaA rate decrease was observed as compared to pre-CPB values. - 39% and 44% reductions of AFXaA levels in comparison to the expected levels in patients with normal or alterad renal function, respectively.	Cytosorb^®^ cartridge in the CPB was safe and associated with rapid correction of Apiyaban-associated anticoagulation.
Buonocore, et al. (2022) [15]	Observational Study: Case report	In-Vivo	81-year-old male patient, haemodynamic unstable, with prosthetic aortic valve endocarditis on apixaban therapy.	-Direct measurement of pre-adsorber inlet and post-adsorber outlet apixaban plasma levels showed a rapid and sustained decrease of the drug through the adsorber.	Cytosorb^®^ proved to be effective for removal of apixaban in emergency surgery setting by direct measurements of drugs levels before and during CPB circulation.
Hassan, et al. (2022) [16]	Observational Study: Case-Control	In-Vivo	25 patients undergoing cardiac surgery who were also on concurrent therapy with apixaban; Control Group (*n*=12) and Cytosorb^®^ Group (n=13).	Cytosorb^®^ group: -No bleeding events and no repeat-thoracotomies occurred; Post-op 24h volume was significantly lower (510±152 vs. 893±579 ml, p=0.03); -No need of DDAVP (0 vs 10±13.6 mg, *p*=0.01).	Use of hemadsorption in patient on apixaban undergoing emergent cardiac surgery, was feasible and safe. Compared to control group, bleeding complications did not occur and the need for 24 h chest-tube-drainage and 1-deamino-8-d-arginine-vasopressin (DDAVP) to achieve hemostasis, was significantly lower on Cytosorb^®^ group.
Dalmastri, et al. (2023) [17]	Observational Study: Case-report	In-Vivo	82-year-old male patient, with AKI in the context of severe bilateral hydroureteronephrosis and other comorbidities, on apixaban therapy due to atrial fibrillation.	-After 2h30 minutes of CRRT with Cytosorb^®^, there was a good reduction of apixaban from 139 to 72 ng/ml (reduction rate of 48.2%) registered. This allowed for an easy placement of bilateral nephrostomies without complications.	Combined treatment with CRRT and Cytosorb^®^ was associated with the rapid and effective removal of Apixaban.

The retrieved studies encompassed various designs, including an experimental in vitro investigation and observational cases studies. Notably, an experimental study examined the reduction of apixaban concentration in an in vitro setting using Cytosorb®. Their results revealed a substantial decrease in apixaban levels after 30 minutes of adsorption, suggesting the potential of this device in reducing apixaban concentrations [14] .

Complementing these findings, observational case studies, presented instances of patients, particularly elderly individuals, undergoing emergent surgeries or dialysis while on apixaban therapy [6, 15, 17]. In each case, the utilization of Cytosorb® demonstrated a reduction in apixaban concentration, indicating its potential role in managing anticoagulation during critical interventions.

Furthermore, the case-control study investigated the impact of Cytosorb® on patients undergoing cardiac surgery while on concurrent apixaban therapy. Noteworthy outcomes include a significant reduction in bleeding events, lower postoperative volumes, and decrease requirements for DDAVP in the Cytosorb® group compared to controls. This study suggests a potential benefit of Cytosorb® in improving hemostatic outcomes during cardiac surgery in patients on apixaban [16].

While these findings collectively suggest a positive trend in the use of Cytosorb® in managing apixaban-associated scenarios, it is essential to acknowledge certain limitations within the existing literature, with the majority of studies being case reports. Moreover, the absence of randomized controlled trials introduces uncertainty regarding causality and necessitates caution in interpreting the observational effects.

However, our brief literature review indicates a growing body of evidence suggesting a potential benefit of Cytosorb® in reducing apixaban concentrations and improving hemostasis in patients undergoing surgeries or dialysis while on apixaban therapy.

It is known that the gold standard to measure plasma apixaban concentration, like other drugs, is mass-spectrometry. However, this technique is not viable for real-time use during emergency cardiac surgery. The anti-Xa test is a common method to measure apixaban concentration. Although is it known that during extracorporeal circulation, apixaban levels are not interpretable due to the indirect inhibition of FXa by heparin, with the administration of protamine, the values become valid, as after neutralization of heparin, the test can be interpreted as the apixaban activity.

In our patient, in line with the existing literature, AFXaA also decreased between the beginning and the end of the surgical intervention. A reduction of 63.7% was recorded between the patient’s admission to the operating room (AFXaA=121 ng/l) and admission to the intensive care unit (AFXaA=44 ng/l). This result can be supported by the postoperative 24h chest-tube-drainage, which was only 280 ml.

We also observed an improvement in liver markers (i.e, alkaline phosphatase and GGT) between the two assessment points, which is also supported by published literature [18].

Regarding limitations, it’s important to note that despite the rapid weaning of vasoactive drugs and the small volume recovered by drains, the stabilization of hemodynamics and coagulopathy cannot be solely attributed to the use of Cytosorb^®^. Aggressive administration of blood products and coagulation factors including fresh plasma, fibrinogen, platelets, prothrombin complex concentrate and recombinant factor VIIa was also necessary.

The measurement of apixaban blood concentration should ideally have been conducted at various stages of the surgery to demonstrate the progressive reduction of this drug during therapy. However, the urgent nature of our case precluded the possibility of conducting this type of analysis.

Another limitation worth noting relates to the monitoring of ACT using the Hemochron system (Hemochron^®^ Signature Elite System, Werfen, Bedford, USA). Although this device provides a quick ACT result in a single measurement, its reliability in assessing the adequacy of heparin anticoagulation monitoring for CPB is questionable. The use of devices like the Hemostasis Management System Plus (HMS^®^, Medtronic, Minneapolis, USA) with simultaneous dual measurements could address this issue; however, we do not have this equipment in our institution.

It is important to emphasize that, although the emergent nature, as well as the advanced age and health status, of our case may pose challenges for extended follow-up, we recognize the importance of considering the long-term implications of hemoadsorption in future research endeavours.

In conclusion, this case demonstrates that intraoperative hemoadsorption with Cytosorb^®^ may be a safe and feasible approach, increasing the elimination of apixaban in patients undergoing emergency cardiac surgery with CPB, resulting in a reduced risk of hemorrhagic complications.

## Data Availability

No datasets were generated or analysed during the current study.

## Abbreviations

CPB: Cardiopulmonary bypass
SIRS: Systemic Inflammatory Response Syndrome
ICU: Intensive Care Unit
NOACs: Novel Oral Anticoagulants
FXa: Factor Xa
HFrEF: Heart failure with reduced ejection fraction
NYHA: New York Heart Association
CT: Computed Tomography
ACT: Activated Clotting Time
AFXaA: Apixaban-Specific anti-factor Xa activity
CRRT: Continuous Renal Replacement Therapy
